# Exploring food insecurity and obesity in Dutch disadvantaged neighborhoods: a cross-sectional mediation analysis

**DOI:** 10.1186/s12889-020-08611-x

**Published:** 2020-04-28

**Authors:** Laura A. van der Velde, Claire J. Nyns, Marije D. Engel, Judith E. Neter, Irene M. van der Meer, Mattijs E. Numans, Jessica C. Kiefte-de Jong

**Affiliations:** 1 Department of Public Health and Primary Care / LUMC-Campus The Hague, Leiden University Medical Centre, The Hague, The Netherlands; 2 Department of Health Sciences, Faculty of Science, Vrije Universiteit Amsterdam, Amsterdam Public Health Research Institute, Amsterdam, The Netherlands; 3 Department of Epidemiology, The Hague’s Public Health Department (GGD Haaglanden), The Hague, The Netherlands; 4 Department of Epidemiology, Erasmus University Medical Centre, Rotterdam, The Netherlands

**Keywords:** Food insecurity, Overweight, Obesity, Diet quality, Mediation

## Abstract

**Background:**

Food insecurity is related to risk of adverse health outcomes such as obesity, but the explanatory factors underlying this association are still unclear. This study aimed to assess the association between food insecurity and obesity, and to explore potential mediation by sociodemographic and lifestyle factors.

**Methods:**

This cross-sectional study was conducted among 250 participants in a deprived urban area in the Netherlands. Data on sociodemographic and lifestyle factors, food insecurity status and diet quality were collected using questionnaires. Diet quality was determined based on current national dietary guidelines. BMI was calculated from self-reported height and weight. Regression analyses were performed to explore the association between food insecurity and BMI status. Mediation analyses were performed to estimate the total-, direct-, and indirect effect and proportion of total effect mediated of the food insecurity-obesity association.

**Results:**

The overall prevalence of food insecurity was 26%. Food insecurity was associated with obesity (OR = 2.49, 95%CI = 1.16, 5.33), but not with overweight (OR = 1.15, 95%CI = 0.54, 2.45) in the unadjusted model. The food insecurity-obesity association was partially mediated by living situation (proportion mediated: 15.4%), diet quality (− 18.6%), and smoking status (− 15.8%) after adjustment for other covariates.

**Conclusions:**

The findings of this study suggest an association between food insecurity and obesity. Living situation, diet quality and smoking status explained part, but not all, of the total association between food insecurity and obesity. Future longitudinal studies are warranted to examine the temporal order of the food insecurity-obesity association and potential mediators in this relationship. In addition, food insecurity and its potential consequences need to be taken into account in obesity prevention programs and policies.

## Background

Food security is defined by the Food and Agriculture Organization (FAO) as “physical and economic access to sufficient, safe and nutritious food that meets dietary needs and food preferences for an active and healthy life” [1]. Initially, most attention regarding food insecurity was focused on low-income countries. However, emerging evidence suggests that food insecurity is also a public health concern facing middle-, and high-income countries [2]. Nevertheless, to date the scientific knowledge on food insecurity in Europe is limited and no clear consensus is reached about the prevalence of food insecurity and its causes and solutions [3]. Specifically, in the Netherlands few studies have focused on the prevalence of food insecurity, especially among community-dwelling subjects. A previous study by Neter et al. (2014) found a food insecurity prevalence of 70% among adult Dutch food bank recipients [4]. Although the latter target group is a selection of extremely disadvantaged individuals, poverty rates are monitored regularly in the Netherlands and indicate that more than 5% of the Dutch population have an income below the basic needs limit, which includes only minimal expenses to cover fundamental needs like food, clothing and housing [5]. Poverty rates are highest in crowded urban districts in the Netherlands [5]. In particular, single-parent households with children below 18 years of age, and people with a non-Western migration background are more vulnerable to poverty [5, 6]. It is therefore reasonable to expect that other disadvantaged groups in the Netherlands, for example those that are not fully eligible to access food bank services, might also be affected by food insecurity and its consequences.

Extensive evidence suggests that food insecurity is related to risk of chronic diseases in adults [7–10], and poorer health, growth and development in the young [11, 12], emphasizing that families with children are particularly vulnerable to the consequences of food insecurity. Although it seems counterintuitive, several studies have found a positive association between food insecurity and obesity in developed countries, particularly among adult women, whereas mixed evidence is found for this association among men and children as well as in developing countries [13–15]. A factor that might explain this association is altered food choices that lead to energy-dense but lower quality diets, as a lower diet quality is related to both food insecurity and obesity [16]. Healthier foods are generally more expensive than unhealthy foods, which might act as a barrier for low-income families to adopt healthier dietary patterns [17]. Studying the factors that might explain the association between food insecurity and obesity is important for public health, since obesity increases the risk of several diseases and other adverse health effects [15, 18]. We therefore assessed the association between food insecurity and obesity among disadvantaged Dutch families, and explored potential mediation by other risk factors for obesity, such as lifestyle factors and social situations.

## Methods

### Study design and study population

This cross-sectional study was conducted in four disadvantaged neighborhoods in the Dutch city The Hague. These neighborhoods were selected based on predefined criteria used by the Dutch Government to identify disadvantaged neighborhoods in the Netherlands, which combined normative data on the socioeconomic position of the households living in the neighborhood and the quality of the neighborhood (i.e. socioeconomic and physical disadvantages), and residents’ opinions on living quality regarding the neighborhood and its residents [19]. Participants were eligible for the study if they (1) were living in or near one of the four selected disadvantaged neighborhoods, (2) were 18 years of age or older, and (3) had at least one child below 18 years of age living at home. Only one parent per household could participate. Participants were recruited between April 2017 and June 2018 by actively approaching potential participants at various public places (e.g. community centers, (pre) schools, community events, swimming pools, and general practices). The study was approved by the Medical Ethics Committee of Leiden University Medical Centre (P17.164).

### Data collection

Data was collected using paper-based or online questionnaires completed by the participants. Most participants completed the questionnaire and informed consent form at the site of recruitment immediately after being invited to the study. Questionnaires were available in the Dutch, English and Turkish language. If participants had difficulty reading or writing, they were offered help completing the questionnaire. If participants provided contact information, they were contacted by phone or e-mail to complement missing data from their questionnaire if applicable.

### Food insecurity status assessment

Household food insecurity status was assessed using the 18-item United States Department of Agriculture Household Food Security Survey Module (USDA HFSSM) [20]. This original survey was translated from the English to the Dutch language based on the translation used in the Dutch study of Neter et al. (2014) which applied the translation and back-translation technique [4]. The survey consists of questions about conditions and behaviors that are characteristic for households having difficulty meeting basic food needs, with the past 12 months as reference period. Affirmative responses to these questions were summed and resulted in a continuum of food insecurity status ranging from 0 to 18, which can be divided into four categories: (1) high food security (0 affirmative responses), (2) marginal food security (1–2 affirmative responses), (3) low food security (3–7 affirmative responses), and (4) very low food security (8–18 affirmative responses) [20]. Range (1) and (2) were categorized as ‘food secure’ (FS), and range (3) and (4) were categorized as ‘food insecure’ (FI), according to the USDA standards [21].

### Dietary assessment and construction of the diet quality scores

Dietary intake was assessed using the Dutch Healthy Diet Food Frequency Questionnaire (DHD-FFQ) [22]. The DHD-FFQ is a short questionnaire comprising 25 questions representing 34 food items, with the previous month as reference period, measuring adherence to Dutch dietary guidelines [22]. We constructed diet quality scores based on the Dutch dietary guidelines on food intake and food choices as indicated by the Health Council of the Netherlands [23] and the Netherlands Nutrition Centre [24]. In this study we present two diet quality score variants: a total diet quality score (TOT-Diet score) and a financially-sensitive diet quality score (FIN-Diet score) (Table 1). The TOT-Diet score included 6 components: vegetables, fruit, fish, bread, oils and fats, and sweet and savory snacks; the FIN-Diet score included 3 components: vegetables, fruit, and fish. We developed the FIN-Diet score in addition to the TOT-Diet score because an adequate intake of vegetables, fruit and fish is important for health, because these components are relatively expensive, and intake may be particularly dependent on financial resources [25, 26]. For each component, a minimum score of 0 and a maximum score of 10 could be obtained, resulting in a total diet quality score ranging from a theoretical minimum of 0 to a theoretical maximum of 30 for the FIN-Diet score and a theoretical maximum of 60 for the TOT-Diet score, with higher scores indicating better adherence to the dietary guidelines (Table 1).

**Table 1:** Diet quality score components, dietary guidelines and scoring per component

Component	Recommendations by the Health Council of the Netherlands and/ or the Netherlands Nutrition Centre	% contribution to component score	Units	Score		
				0	5	10
Vegetables^a,b^	Eat at least 200 grams of vegetables daily	100	Grams/ d	0	*Continuous*	≥200
Fruit^a,b^	Eat at least 200 grams of fruit daily	100	Pieces/ d	0	*Continuous*	≥ 2
Fish^a,b^	Eat one serving of fish weekly, preferably oily fish	50	Servings/ w	0	<1	≥ 1
		50	-	No fish consumed	Lean or both lean and fatty fish	Mostly fatty fish
Bread^b^	Replace refined cereal products by whole-grain products	50	-	Mostly white bread	Both white and brown/ whole-grain bread	Mostly brown/ whole-grain bread
	Women: 4-5 brown/ whole-grain sandwiches daily	50	Sandwiches/ d	0	*Continuous*	≥ 4
	Men: 6-8 brown/ whole-grain sandwiches daily		Sandwiches/ d	0	*Continuous*	≥ 6
Oils and fats^b^	Replace butter, hard margarines and cooking fats by soft margarines, liquid cooking fats, and vegetable oils	50	-	Butter, hard margarines	Both butter, hard margarines and oils and soft margarines	Oils and soft margarines
		50	-	Butter on bread or bread is not buttered at all	Semi-skimmed butter or hard margarine on bread	Diet margarine on bread
Sweet and savory snacks^b^	For products outside the Wheel of Five: choose an item from the daily selection no more than three to five times per day, and something from the weekly selection no more than three times a week	25	Sweet snacks (larger serving)/ w	≥ 3	<1 to 2	Not consumed
		25	Sweet snacks (small serving)/ d	> 3	*Continuous*	Not consumed
		25	Savory snacks (larger serving)/ w	≥ 3	1 to 2	Not consumed
		25	Savory snacks (small serving)/ d	> 3	*Continuous*	Not consumed

^a^Components included in the FIN-Diet score: vegetables; fruit; and fish

^b^Components included in the TOT-Diet score: vegetables; fruit; fish; bread; oils and fats; and sweet and savory snacks

### Sociodemographic and lifestyle factors

Sociodemographic and lifestyle information was collected, including age or date of birth, sex, height, weight, gross monthly household income, household composition, marital status, educational level, country of birth of the participant and their parents, employment status, smoking status, food bank use, religion, pregnancy status, and physical activity. Self-reported general health status was assessed using a 5-point Likert scale ranging from excellent to poor, and dichotomized into ‘good-to-excellent’ and ‘fair-to-poor’. Age was calculated by extracting the date of birth of the participant from the date on which the questionnaire was completed and was presented in years. If the date of birth of the participant was not available, we used their self-reported age in years. Body Mass Index (BMI, kg/m^2^) of the participants was calculated from their self-reported weight and height, and classified into underweight (BMI < 18.5 kg/m^2^), normal weight (BMI 18.5–25 kg/m^2^), overweight (BMI 25–30 kg/m^2^) and obese (BMI ≥ 30 kg/m^2^), using the WHO cut-off points [25]. Only 1.5% of the participants were classified as underweight and the lowest BMI was 17, therefore they were included in the normal weight category.

Gross monthly household income was dichotomized into above or below the Dutch basic needs budget [5], which was calculated taking into account the household size and composition according to the method drawn up by Statistics Netherlands [27]. Household composition was presented as the adult/child ratio (number of adults divided by the number of children). Marital status was used to derive the living situation: single or married/partner. The educational level categories were based on the International Standard Classification of Education (ISCED) 2011 [28], and dichotomized into a low educational level (≤ISCED 2) and higher educational level (≥ISCED 3). Migration background of the participants was based on the country of birth of the parents: if one parent was born outside of the Netherlands, the country of birth of that parent determined the participants’ migration background. If both parents were born abroad, the country of birth of the mother determined the participants’ migration background [29]. Physical activity (i.e. days per week and minutes per day being moderately active) was assessed as part of the DHD-FFQ [22].

### Potential mediating variables and covariates

To evaluate the magnitude of disparity in obesity due to food insecurity that would remain if an intermediate or downstream determinant is changed, we selected various potential mediating variables on the basis of the literature [13, 30, 31]. The association between food insecurity and weight was previously found to be mediated by lifestyle health behaviors like diet quality and physical activity [30]. To illustrate, food insecurity might influence weight through changing physical activity and therefore physical activity is considered a potential mediator. For example, experiencing food insecurity may decrease physical activity (i.e. through symptoms of fatigue due to reduced dietary quality and potential deficiencies or limited financial possibilities to engage in sports). In turn, a decrease in physical activity could increase obesity prevalence through an altered energy expenditure [30]. Further, living situation and stressors (which might trigger unhealthy coping mechanisms like smoking) were previously indicated as potential mediators in this relationship [13, 31]. As a result, the following variables were considered as potential mediating variables that may explain the food insecurity-obesity association: living situation, physical activity, household composition, smoking status, self-reported general health status, FIN-Diet score, and TOT-Diet score. A preliminary theoretical model and explanation of these associations is shown in Additional Figure 1. The individual characteristics age, sex, household income, educational level, and migration background were considered as additional covariates.

### Statistical analysis

Subject characteristics, food insecurity status, general health status, diet quality, and BMI status were described as median (interquartile range, IQR) for continuous variables and frequencies and percentages for categorical variables. The association between food insecurity and BMI status was evaluated using multinomial logistic regression. Four models were presented: a crude model; and models adjusted for basic characteristics, socioeconomic status (SES) and lifestyle factors.

Mediation analyses were performed for the continuous food insecurity status score-obesity association, with living situation, physical activity, household composition, smoking status, self-reported general health status, FIN-Diet score and TOT-Diet score as potential mediating variables. All potential mediating variables were tested step by step. We used Stata’s binary mediation program to estimate the standardized total-, direct-, and indirect effect and the proportion of total effect mediated of each of the above mentioned potential mediators separately, both crude and controlling for covariates. Standard errors and confidence intervals were obtained using the bootstrapping method (1000 repetitions) [32]. We presented bias-corrected 95% confidence intervals to account for non-normal distributed data, as these are considered most accurate [33, 34]. The indirect effect (i.e. the mediated association) was estimated using the product of coefficients approach [32] (Additional document 1). The indirect effect reflects the extent to which the independent variable (food insecurity status) is associated with the potential mediating variable, and the extent to which the potential mediating variable is associated with the dependent variable (obesity). Mediation was assumed to have occurred when the indirect effect was statistically significantly different from zero. Complete mediation occurred when the direct effect (i.e. the association between the independent variable and the dependent variable when controlling for the mediating variable) became non-significant, indicating that the total effect (i.e. the sum of the indirect and direct effect) was completely explained by the mediating variable. Partial mediation occurred when both the indirect and direct effect were statistically significantly different from zero, indicating that the mediating variable explained part, but not all, of the total association. If the direct effect is opposite in sign to the indirect effect, this is referred to as inconsistent mediation [35].

Multiple imputation was used to reduce potential attrition bias associated with missing data including all analysis variables, assuming that missing values were missing at random. Ten imputed datasets were generated using fully conditional specification (Markov chain Monte Carlo method) with a maximum of 10 iterations. Predictive mean matching was used for not-normally distributed variables, logistic regression models for categorical variables. Further details of the multiple imputation are presented in Additional Table 1. Because participant characteristics were similar in the imputed and unimputed data, pooled results after the multiple imputation were presented (Additional Table 2).

Mediation analyses were conducted using Stata version 14.0 (StataCorp,2015. Stata Statistical Software. College Station, TX:StataCorp LP). All other statistical analyses were performed using SPSS version 25.0 (IBM Corp., 2012, Armonk, NY). A two-sided *P*-value of 0.05 was considered statistically significant.

## Results

### Participant characteristics

In total, 250 participants completed the questionnaire, of whom 8 were excluded (due to not having children below 18 years of age (*n* = 7), and (*n* = 1) for living outside the study area), resulting in a population of analysis of 242 participants. The overall prevalence of food insecurity was 26.0%; 18.2% of the participants experienced low food security and 7.8% experienced very low food security (Table 2).

**Table 2:** Food insecurity status in four categories and total food secure and food insecure participants

Food insecurity status	n (%)
High food security	127 (52.5)
Marginal food security	52 (21.5)
Total food secure	179 (74.0)
Low food security	44 (18.2)
Very low food security	19 (7.8)
Total food insecure	63 (26.0)

Compared to food secure (FS) participants, food insecure (FI) participants more often had an income below the basic needs budget, had a lower educational level, and were less often currently employed. FI participants more often had a non-Western migration background, and were more often Christian and less often Islamic compared to FS participants (Additional Table 3). Compared to FS participants, FI participants were more often single parents and current smokers. Self-reported general health status was poorer among FI participants, as they reported fair-to-poor health more than twice as often as FS participants (Additional Table 4). The average TOT-Diet score and FIN-Diet score varied across food insecurity status categories, with the lowest scores obtained by participants with a very low food security status. Overall, FI participants had a slightly lower median TOT-Diet score and a 4.6 points lower FIN-Diet score compared to FS participants (Additional Tables 4 and 5). Only the components fruit, vegetables, and fish differed statistically significantly between FS and FI participants, with FI participants showing lower scores (Additional Table 5). Additional Table 6 shows differences in component and total diet scores for obese and non-obese participants.

### Food insecurity and BMI status

**Fig. 1:**
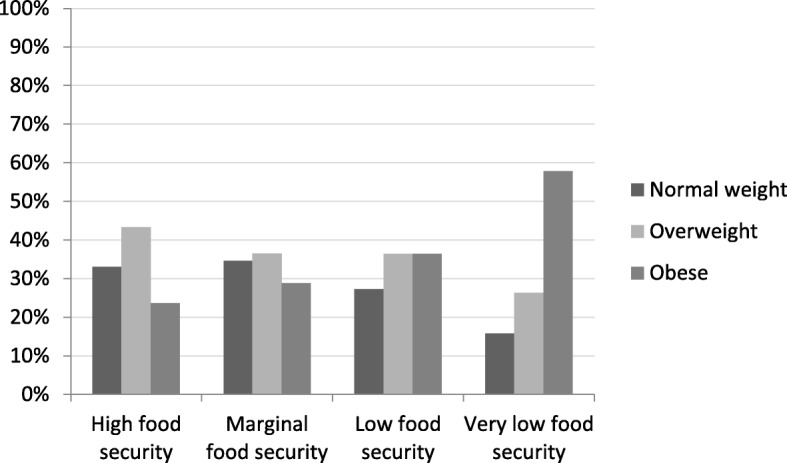
BMI status across food insecurity status categories

Obesity prevalence markedly increased with an increasing food insecurity status; obesity prevalence increased from 23.6% among participants experiencing high food security to 57.9% among participants experiencing very low food security (Fig. 1). Overall, 25.1% of the FS participants were obese, while 42.9% of the FI participants were obese.

Food insecurity was associated with obesity, but not with overweight. FI participants were 2.49 (95%CI = 1.16,5.33) times more likely to be obese than FS participants. Controlling for basic characteristics, SES and lifestyle factors, the odds ratio was similar but not statistically significant (Table 3).

**Table 3:** Associations between food insecurity status and BMI status

	Overweight		Obesity	
	OR (95%CI)	***p***-value	OR (95%CI)	***p***-value
Crude model	1.15 (0.54, 2.45)	0.721	2.49 (1.16, 5.33)	0.019*
Model 1: basic characteristics adjusted	0.78 (0.34, 1.79)	0.559	1.94 (0.84, 4.51)	0.123
Model 2: SES adjusted	0.80 (0.34, 1.89)	0.610	1.57 (0.65, 3.79)	0.312
Model 3: lifestyle factors adjusted	1.15 (0.46, 2.85)	0.769	2.51 (0.98, 6.48)	0.056

### Explaining the association between food insecurity and obesity

**Fig. 2:**
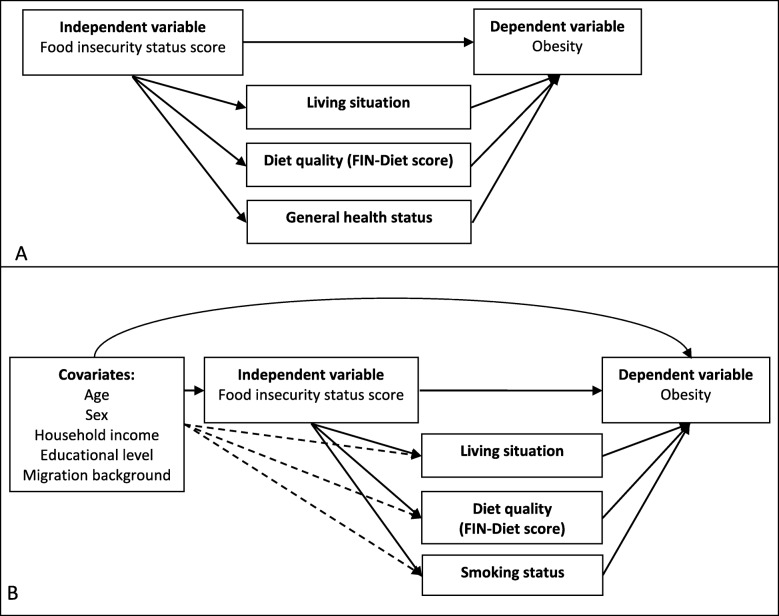
The association between food insecurity status score and obesity and its partial mediators

The unadjusted mediation analyses showed that the food insecurity-obesity association was partially mediated by living situation and general health status (consistent mediation). Diet quality (FIN-Diet score) was an inconsistent partial mediator. The proportion of total effect mediated ranged between 15.3 and 19.1% for all described mediators (Table 4, Fig. 2, Additional Table 7). After adjustment for covariates, living situation remained a consistent partial mediator and the FIN-Diet score remained an inconsistent partial mediator. Further, smoking status was an inconsistent partial mediator after adjustment (Table 4, Fig. 2, Additional Table 8). Additional Tables 7 and 8 show mediation statistics for all tested potential mediators.

**Table 4:** Mediation statistics of statistically significant mediators of the food insecurity status score-obesity association

	Unadjusted			Adjusted^**a**^		
	Indirect effect		Proportion of total effect mediated	Indirect effect		Proportion of total effect mediated
	Estimate	95% CI^b^	%	Estimate	95% CI^b^	%
Mediators						
Living situation	0.037*	0.0073, 0.096	15.3	0.036*	0.0013, 0.11	15.4
Diet quality (FIN-Diet score)	−0.041*	−0.11, − 0.0012	−17.7	−0.042*	− 0.10, − 0.0019	−18.6
General health status	0.044*	0.00089, 0.11	19.1			
Smoking status				−0.034*	−0.11, − 0.00034	−15.8

*CI* Confidence Interval

*Statistically significant (*p* < 0.05)

^a^Adjusted for age, sex, household income, educational level, and migration background

^b^Bias-corrected

## Discussion

The present study showed that a quarter of the participating disadvantaged families experienced food insecurity. Food insecurity status was associated with obesity in the unadjusted model, while after adjustment similar but non-significant effect estimates were observed. Living situation, diet quality (FIN-Diet score) and smoking status explained part, but not all, of the total association between food insecurity and obesity after adjustment for other covariates.

Our result on food insecurity prevalence is agreement with a large global study on food insecurity and mental health, which found approximately the same food insecurity prevalence across 39 countries in Europe, although that study used a different questionnaire to assess food insecurity [36].

Our results suggest a positive association between food insecurity and obesity. Previous studies imply that gender differences and the economic development level of a country are important factors in this association, since a positive association between food insecurity and obesity is particularly evident among women in developed countries, whereas mixed evidence for an association has been found among men and children and among populations living in developing countries [15, 16]. For example, a recent systematic review and meta-analysis by Moradi et al. [15] indicates that food insecurity increases the risk of obesity, but not underweight nor overweight among adults in high-income countries. In our study, obesity prevalence increased considerably with increasing food insecurity status. Previous studies also found a linear association between food insecurity status and obesity prevalence, whereas other studies found a U-shaped association [13].

Regarding gender differences, earlier literature suggests that the positive association between food insecurity and obesity is especially evident in women [13–15], which is comparable to our results since the study population consisted predominantly of women. Because of this uneven gender distribution we were unable to further explore gender differences in our study. However, Martin & Lippert (2012) have elaborated on this and suggest that gender differences in the association between food insecurity and obesity might be attributed to motherhood (and the social role of the mother to feed the family [37]); mothers might adopt unhealthy strategies in order to protect their children when experiencing household food insecurity, which may increase their risk of an unhealthy weight [38].

Notably, the results of our study suggest a positive association between food insecurity status and obesity, but not between food insecurity and overweight. Previous literature also suggests stronger associations between food insecurity and obesity than with overweight [39], which might be due to a larger heterogeneity in factors and situations leading to overweight (such as age related factors), whereas underlying causes of obesity might be more severe and specific (such as mental health issues, stress, and experiencing food insecurity). For example, food insecurity may cause temporal involuntary food intake restrictions due to insufficient resources to access food, followed by a period of excessive food intake when food becomes available again, a phenomenon known as the feast-famine cycle [14]. Such a disruptive eating pattern can lead to metabolic alterations and eventually result in obesity [14].

The explanatory factors underlying the association between food insecurity and obesity are not yet completely established [15]. By exploring the mediating role of several risk factors for obesity, our study provides additional insight into this complex association. We identified diet quality (the FIN-Diet score) and smoking status as inconsistent partial mediators, and living situation and general health status as partial mediators of the association between food insecurity and obesity.

While food insecurity is clearly associated with obesity and a lower diet quality [16], how food insecurity, diet quality and obesity interrelate is less clear however. One study found no evidence for a mediating role of diet quality in the association between food insecurity and weight [40]. Another study suggested fruit and vegetable consumption as a potential mediator in the association between food insecurity and obesity [37]. In our study the food insecurity-obesity association was inconsistently partially mediated by the FIN-Diet score and not statistically significantly mediated by the TOT-Diet score, implying that diet quality did not fully explain the association between food insecurity and obesity. The relatively higher cost of a diet high in fruit, vegetables and fish might play a role in the stronger impact that was found for the FIN-Diet score compared to the TOT-Diet score [17]. Strikingly, similar results were observed when controlling for income, which suggests that income itself cannot fully explain these findings and that other constructs such as financial capacity or financial stress may be more important. Previous literature also indicates an association between perceived stress and unhealthy eating behaviors, such as emotional eating and haphazard meal planning, which eventually may lead to obesity [41–43].

Smoking status partially and inconsistently mediated the food insecurity-obesity association, indicating that smoking had an overall suppressing effect on the association between food insecurity and obesity. Food insecure persons may smoke more than their food secure counterparts as a way to cope with stressors such as financial stress and as a way to suppress their appetite, while smoking in turn might lead to a lower body weight due to an increased energy expenditure and reduced food intake [44, 45].

Living situation (specifically being single as opposed to having a partner) was also found to partially mediate the food insecurity-obesity association. Food insecurity and the higher stress levels associated with it may lead to lower marital satisfaction and thereby decreased relationship maintenance [46, 47]. In turn, single parents (specifically single mothers) are not only more at risk of food insecurity, but the consequences of food insecurity on their weight are also greater compared to partnered women [38]. This could be a reflection of the difficult task of being the sole provider in the household while also being responsible for child care [38].

Finally, general health status partially mediated the food insecurity-obesity association through poorer health. In line with previous studies, we found that food insecurity was associated with poorer health [48] and poorer health was associated with obesity [49]. The mediating role of general health status in this association was mainly explained by other sociodemographic factors.

A strength of our study was the assessment of many sociodemographic and lifestyle factors, which enabled an extensive description of the study population, adjustment of the analyses and exploration of several potential mediators. Food insecurity is an elusive concept involving many factors, and many different indicators have been described in literature [50]. We used the widely accepted 18-item USDA Household Food Security Survey Module (USDA HFSSM) to assess food insecurity status, which is regarded as the gold standard for Western countries [20, 51]. It should, however, be noted that the USDA HFSSM and our translation have not yet been validated specifically for the Dutch population, which may have led to misclassification in our study. However, these effects are assumed to be limited, as the USDA HFSSM has previously been adapted for use in various cultures and languages and generally shows to be a valid tool for the assessment of food insecurity status [52–54]. In addition, a recent literature review showed that strategies to cope with food insecurity are similar across different ethnic/racial groups, but more research on the ethnic differences in perception of food insecurity and coping strategies is needed [55].

Further limitations of this study should also be considered when interpreting our results. Some measures were supposed to reflect the household situation (e.g. income and food insecurity status). Because data were reported by one person they may not reflect the views of other family members. The data were self-reported which may have led to misclassification. For BMI this may have led to an underestimation of the actual prevalence of overweight and obesity [56], indicating that the obesity prevalence might be even higher than found in our study. Also, we used validated measures for dietary intake [22] and general health status [57], thus we assume that misclassification bias had a limited effect on our main findings.

The reference period for the food insecurity assessment was 12 months, whereas the reference period for the dietary intake assessment was only 1 month. These differing reference periods may explain the partial mediation by dietary quality in the association between food insecurity and obesity that was observed in the current study: a stronger effect might have been observed when the reference periods were matched because this would have reflected a more direct association between food insecurity status and dietary quality. However, a previous study by Huddleston-Casas et al. (2009) showed a strong correlation between food insecurity scores over a period of 2 years [58] indicating that food insecurity status is relatively stable over time. Therefore, the effect of this longer reference period is expected to have a limited effect on the association between food insecurity and diet quality and the validity of our conclusions.

The short FFQ used in our study to assess dietary intake and compute diet quality scores contained only a limited range of foods. Although the DHD-FFQ could adequately provide an approximate ranking of subjects according to their diet quality, the DHD-FFQ is most applicable to Dutch eating patterns and to a lesser extent to non-Dutch eating patterns [22]. Also, this short FFQ did not enable a detailed assessment of nutrient intakes and therefore our diet quality scores could not be validated by relating them to nutrient adequacy [59], which would have been a valuable contribution.

Our study was cross-sectional and therefore no causal relations could be established. This is especially important for the mediation analyses, as this precludes any conclusions regarding the nature of the observed associations. It should further be noted that conducting mediation analyses using cross-sectional data and a binary outcome has been criticized by others [60]. However, to overcome limitations associated with cross-sectional data and binary outcomes variables, we used the product of coefficients approach as recommended for this situation [61]. Also, we did not aim for establishing causal pathways between food insecurity and obesity but rather aimed to evaluate the magnitude of disparity in obesity due to food insecurity that would remain if an intermediate or downstream risk factor is changed. Future longitudinal studies will be needed to examine the temporal order of the food insecurity-obesity association and potential mediators in this relationship.

## Conclusion

The findings of this study suggest an association between food insecurity and obesity. This association is partially mediated by living situation, and inconsistently (i.e. the direct effect was opposite in sign to the indirect effect) partially mediated by diet quality (FIN-Diet score) and smoking status in disadvantaged Dutch families, indicating that living situation, diet quality and smoking status explained part, but not all, of the total association between food insecurity and obesity. Overall, our findings emphasize the importance of preventing food insecurity to achieve public health goals. Even though the association between food insecurity status and obesity remains complex, our study contributes to a better understanding of how these two public health concerns might be related. However, because major aspects of the association between food insecurity and obesity are still unexplained, future studies are warranted to test other potential mediators such as financial stress, sleep, and other indices of dietary quality, which might guide future prevention programs.

## Supplementary information

**Additional file 1: Figure S1.** Preliminary theoretical model of the food insecurity status-obesity association and its potential mediators. **Document 1.** Example of the Stata do-file used for the mediation analyses. **Table S1.** Details of the multiple imputation process. **Table S2.** Participant characteristics in original and imputed data. **Table S3.** Covariates used in the mediation analyses in the total population and across food insecurity categories. **Table S4.** Potential mediators used in the mediation analyses in the total population and across food insecurity categories. **Table S5.** Diet quality (component) scores, for the total study population and split by food insecurity status. **Table S6.** Diet quality (component) scores, in total and split by food insecurity status and obesity status. **Table S7.** Mediation statistics of the food insecurity status score-obesity association and all potential mediators (unadjusted). **Table S8.** Mediation statistics of the food insecurity status score-obesity association and all potential mediators (adjusted).

## Data Availability

The datasets used and analyzed for the current study are available from the corresponding author on reasonable request.

## Abbreviations

BMI: Body Mass Index
CI: Confidence Interval
DHD: Dutch Healthy Diet
FAO: Food and Agriculture Organization
FFQ: Food Frequency Questionnaire
FS: Food secure
FI: Food insecure
HFSSM: Household Food Security Survey Module
ISCED: International Standard Classification of Education
IQR: Interquartile range
FIN-Diet score: Financially-sensitive diet quality score
TOT-Diet score: Total diet quality score
USDA: United States Department of Agriculture
SES: Socioeconomic status
