# Supplementary material for: Exploring food insecurity and obesity in Dutch disadvantaged neighborhoods: a cross-sectional mediation analysis
Source: BMC Public Health. 2020 Apr 28;20:569. doi: 10.1186/s12889-020-08611-x (PMC7189681; doi:10.1186/s12889-020-08611-x)
Supplement: Supplementary file 1 — Additional file 1: Figure S1. Preliminary theoretical model of the food insecurity status-obesity association and its potential mediators. Document 1. Example of the Stata do-file used for the mediation analyses. Table S1. Details of the multiple imputation process. Table S2. Participant characteristics in original and imputed data. Table S3. Covariates used in the mediation analyses in the total population and across food insecurity categories. Table S4. Potential mediators used in the mediation analyses in the total population and across food insecurity categories. Table S5. Diet quality (component) scores, for the total study population and split by food insecurity status. Table S6. Diet quality (component) scores, in total and split by food insecurity status and obesity status. Table S7. Mediation statistics of the food insecurity status score-obesity association and all potential mediators (unadjusted). Table S8. Mediation statistics of the food insecurity status score-obesity association and all potential mediators (adjusted). [file 12889_2020_8611_MOESM1_ESM.docx]

Additional material

Exploring food insecurity and obesity in Dutch disadvantaged neighborhoods: a cross-sectional mediation analysis

**Contents**

[Additional Figure 1 2](#_Toc36018063)

[Additional document 1 3](#_Toc36018064)

[Additional Table 1 4](#_Toc36018065)

[Additional Table 2 5](#_Toc36018066)

[Additional Table 3 6](#_Toc36018067)

[Additional Table 4 7](#_Toc36018068)

[Additional Table 5 8](#_Toc36018069)

[Additional Table 6 9](#_Toc36018070)

[Additional Table 7 10](#_Toc36018071)

[Additional Table 8 11](#_Toc36018072)

Additional Figure 1 Preliminary theoretical model of the food insecurity status-obesity association and its potential mediators^1^

**Dependent variable**

Obesity

**Independent variable**

Food insecurity status

**Covariates:**

Age

Sex

Household income

Educational level

Migration background

**Mediating variables**

Living situation

Physical activity

Household composition

Smoking status

General health status

Diet quality

^1^This preliminary theoretical model of the association between food insecurity status and obesity shows that food insecurity might directly influence obesity status, but might also indirectly influence obesity status through the potential mediating variables Living situation; Physical activity; Household composition; Smoking status; General health status; and/or Diet quality. These potential mediating variables were selected on the basis of the literature (Franklin et al. (2012); Martinez et al. (2019); Hanson et al. (2007)). The covariates Age; Sex; Household income; Educational level; and Migration background were included in the model to ensure that these variables did not confound any of the assessed direct and indirect associations between food insecurity status and obesity.

Additional document 1 Example of the Stata do-file used for the mediation analyses

/* MEDIATION OF FOOD SECURITY - OBESITY RELATIONSHIP */

/* With Food security score as continuous IV */

/*XXX M= Living situation (MaritalStat_STATA) XXX*/

/*Not adjusted for covariates: */

binary_mediation, dv(Obesity_2cat_STATA) iv(Score_FoodSecurity) mv(MaritalStat_STATA)

/*Create CI intervals using boostrapping*/

quietly bootstrap r(indir_1)r(tot_ind) r(dir_eff) r(tot_eff), ///

reps(1000): binary_mediation, dv(Obesity_2cat_STATA) iv(Score_FoodSecurity) mv(MaritalStat_STATA)

estat bootstrap, percentile bc

/*Adjusted for covariates:*/

binary_mediation, dv(Obesity_2cat_STATA) iv(Score_FoodSecurity) mv(MaritalStat_STATA) cv(Leeftijd_totaal Sex_STATA Inkomen_2cat_STATA Opleiding_2cat_STATA Migratie_2cat_STATA)

/*Create CI intervals using boostrapping*/

quietly bootstrap r(indir_1)r(tot_ind) r(dir_eff) r(tot_eff), ///

reps(1000): binary_mediation, dv(Obesity_2cat_STATA) iv(Score_FoodSecurity) mv(MaritalStat_STATA) cv(Leeftijd_totaal Sex_STATA Inkomen_2cat_STATA Opleiding_2cat_STATA Migratie_2cat_STATA)

estat bootstrap, percentile bc

Additional Table 1 Details of the multiple imputation process

|  | **Multiple imputation** |
| --- | --- |
| Software used | IBM SPSS statistics version 25 |
| Imputation method and key settings | Fully conditional specification (Markov chain Monte Carlo method); maximum iterations: 10 |
| No· of imputed data sets created | 10 |
| Variables included in the imputation procedure as both predictor variable as a variable to be imputed | Wijken Leeftijd_totaal Geslacht Lengte Gewicht  Inkomen_BasicNeeds migratieachtergrond Burgerlijke_staat Opleidingsniveau_3cat Religie Voedselbank roken_totaal Aantal_volwassenen_huishouden Aantal_kinderen_huishouden Betaalde_baan_totaal Zwanger |
| Variables added as predictors (not used in the main analyses) of missing data to increase plausibility of missing at random assumption | land_geboren_5cat Land_vader_5cat Land_moeder_5cat  Huidig_betaalde_baan Verleden_betaalde_baan Huidig_Roken Verleden_Roken  Score_FoodSecurity_1 Score_FoodSecurity_2 Score_FoodSecurity_3 Score_FoodSecurity_4 Score_FoodSecurity_5 Score_FoodSecurity_6  Score_FoodSecurity_7 Score_FoodSecurity_8 Score_FoodSecurity_10 Score_FoodSecurity_11 Score_FoodSecurity_12 Score_FoodSecurity_13  Score_FoodSecurity_14 Score_FoodSecurity_15 Score_FoodSecurity_16  Eetgewoonten_1a Eetgewoonten_1b Eetgewoonten_1d Eetgewoonten_1e Eetgewoonten_2a Eetgewoonten_2b Eetgewoonten_3a Eetgewoonten_4a Eetgewoonten_4b Eetgewoonten_5 Eetgewoonten_6 Eetgewoonten_7 Eetgewoonten_8a Eetgewoonten_9a Eetgewoonten_10a Eetgewoonten_11a Eetgewoonten_12a Eetgewoonten_13a  Eetgewoonten_15_roomboterEetgewoonten_15_vet_uit_pakje Eetgewoonten_15_vet_uit_fles Eetgewoonten_15_olie Eetgewoonten_16 Eetgewoonten_17a Eetgewoonten_18a Eetgewoonten_19a Eetgewoonten_20 Eetgewoonten_21a  Eetgewoonten_22a Eetgewoonten_23 Eetgewoonten_24 Lichaamsbeweging_minuten  SF_1 SF_2a SF_2b SF_3a SF_3b SF_4a SF_4b SF_5 SF_6a SF_6b SF_6c SF_7 Gezondheid_hoge_bloeddruk Gezondheid_hoog_cholesterol Gezondheid_Ingreep_hart Gezondheid_hartaanval Gezondheid_asthma Gezondheid_COPD Gezondheid_diabetes Gezondheid_anemie Gezondheid_zonder_problemen_boodschappen |
| Treatment of not normally distributed variables | Predictive mean matching |
| Treatment of binary/categorical variables | Logistic regression models |
| Population | For the multiple imputation we included all adult participants living in (or near) one of the selected neighbourhoods in The Hague with children below 18 years of age living at home (n= 242). |

Additional Table 2 Participant characteristics in original and imputed data

|  | | | | **Missing in original data**  **n (%)** | **Original data**  **n=242** | **Data after Multiple Imputation**  **n=242** |
| --- | --- | --- | --- | --- | --- | --- |
| **Characteristics** | | | |  |  |  |
| Age (y) (median (IQR)) | | | | 13 (5·4) | 37·3 (33·6; 42·4) | 37·5 (33·5; 42·5) |
| Sex (n (%) female) | | | | 3 (1·2) | 209 (87·4) | 211 (87·2) |
| Household income (n (%)) | | | | 28 (11·6) |  |  |
|  | | Below basic needs budget | |  | 145 (67·8) | 159 (65·7) |
|  | | Above basic needs budget | |  | 69 (32·2) | 83 (34·3) |
| Educational level^a^ (n (%)) | | | | 8 (3·3) |  |  |
|  | | Low (≤ISCED 2) | |  | 97 (41·5) | 99 (40·9) |
|  | | Higher (≥ISCED 3) | |  | 137 (58·5) | 143 (59·1) |
| Migration background (n (%)) | | | | 4 (1·7) |  |  |
|  | | Western (including Dutch) | |  | 35 (14·7) | 36 (14·9) |
|  | | Turkish | |  | 47 (19·7) | 48 (19·8) |
|  | | Moroccan | |  | 66 (27·7) | 67 (27·7) |
|  | | Surinamese | |  | 26 (10·9) | 27 (11·2) |
|  | | Other | |  | 64 (26·9) | 65 (26·4) |
| Religion (n (%)) | | | | 25 (10·3) |  |  |
|  | Christianity | | |  | 39 (18·0) | 44 (18·2) |
|  | Islam | | |  | 138 (63·6) | 142 (58·7) |
|  | Not religious/ other | | |  | 40 (18·4) | 56 (23·1) |
| Living situation (n (%)) | | | | 8 (3·3) |  |  |
|  | | Married/ partner | |  | 161 (68·8) | 165 (68·2) |
|  | | Single | |  | 73 (31·2) | 77 (31·8) |
| Household composition (adult/ child ratio) (median (IQR)) | | | | 13 (5·4) | 1 (0·5;1·0) | 1·0 (0·5; 1·0) |
| Employment status (n (%)) | | | | 4 (1·7) |  |  |
|  | | Currently employed | |  | 99 (41·6) | 100 (41·3) |
|  | | Employed in the past | |  | 90 (37·8) | 91 (37·6) |
|  | | Never employed | |  | 49 (20·6) | 51 (21·1) |
| Food Bank use^b^ (n (%) yes) | | | | 6 (2·5) | 7 (3·2) | 17 (7·0) |
| Pregnancy^b^ (n (%) yes among women) | | | | 4 (1·7) | 3 (1·8) | 17 (8·1) |
| Weight status (n (%)) | | | | 12 (5·0) |  |  |
|  | | | Not obese (BMI < 30) |  | 162 (70·4) | 170 (70·2) |
|  | | | Obese (BMI ≥30) |  | 68 (29·6) | 72 (29·8) |
| Weight status (n (%)) | | | | 12 (5·0) |  |  |
|  | | | Normal weight (BMI <25)^c^ |  | 70 (30·4) | 75 (31·0) |
|  | | | Overweight (BMI 25-30) |  | 92 (40·0) | 95 (39·3) |
|  | | | Obesity (BMI ≥30) |  | 68 (29·6) | 72 (29·8) |
| Smoking status (n (%)) | | | | 13 (5·4) |  |  |
|  | | | Current smoker |  | 37 (16·2) | 41 (16·9) |
|  | | | Past smoker |  | 34 (14·8) | 39 (16·1) |
|  | | | Non-smoker |  | 158 (69·0) | 162 (66·9) |
| General health status^b^ (n (%)) | | | | 0 (0·0) |  |  |
|  | | Good to excellent | |  | 149 (74·9) | 149 (74·9) |
|  | | Fair to poor | |  | 50 (25·1) | 50 (25·1) |
| Physical activity (min/ d) (median (IQR) | | | | 0 (0·0) | 8·6 (2·0; 17·1) | 8·6 (2·0; 17·1) |

^a^ISCED 2= Lower secondary education; ISCED 3= Upper secondary education

^b^These questions were added at a later stage during the study and therefore questions that were missing because they were not yet included in the questionnaires are not included in the percentage missing column Correctly missing (n (%)): ‘Food Bank use’ (15 (6·2)), ‘Pregnancy’ (43 (17·8)), ‘General health status’ (43 (17·8))

^c^Only 2 participants were underweight (BMI < 18·5) and they were therefore included in the normal weight category

Additional Table 3 Covariates used in the mediation analyses in the total population and across food insecurity categories

|  | | Total population  (n=242) | High food security (n=127) | Marginal food security (n=52) | Low food security (n=44) | Very low food security (n=19) | Total food secure (n=179) | Total food insecure (n=63) |  |
| --- | --- | --- | --- | --- | --- | --- | --- | --- | --- |
| **Covariates**  n (%) or median (IQR) | |  |  |  |  |  |  |  | Significance^c^ (* = p <0.05) |
| Age (y) (median (IQR))^a^ | | 37·5 (33·5, 42·5) | 37·1 (34·0, 41·5) | 35·3 (31·2, 42·4) | 39·0 (34·0, 44·7) | 39·7 (36·3, 45·5) | 37·0 (32·5, 42·0) | 39·5 (34·9, 45·0) | * |
| Sex | |  |  |  |  |  |  |  |  |
|  | Male | 31 (12·8) | 18 (14·2) | 3 (5·7) | 5 (11·4) | 5 (26·3) | 21 (11·7) | 10 (15·9) |  |
|  | Female | 211 (87·2) | 109 (85·8) | 49 (94·2) | 39 (88·6) | 14 (73·7) | 158 (88·3) | 53 (84·1) |  |
| Household income | |  |  |  |  |  |  |  | * |
|  | Below basic needs budget | 159 (65·7) | 63 (49·6) | 42 (80·8) | 38 (86·4) | 16 (84·2) | 105 (58·7) | 54 (85·7) |  |
|  | Above basic needs budget | 83 (34·3) | 64 (50·4) | 10 (19·2) | 6 (13·6) | 3 (15·8) | 74 (41·3) | 9 (14·3) |  |
| Educational level^b^ | |  |  |  |  |  |  |  | * |
|  | Low (≤ISCED 2) | 99 (40·9) | 42 (33·1) | 24 (46·2) | 23 (52·3) | 11 (57·9) | 65 (36·3) | 34 (54·0) |  |
|  | Higher (≥ ISCED 3) | 143 (59·1) | 85 (66·9) | 29 (55·8) | 21 (47·7) | 8 (42·1) | 114 (63·7) | 29 (46·0) |  |
| Employment status | |  |  |  |  |  |  |  | * |
|  | Currently employed | 100 (41·3) | 69 (54·3) | 14 (26·9) | 11 (25·0) | 6 (31·6) | 83 (46·4) | 17 (27·0) |  |
|  | Currently not employed | 142 (58·7) | 58 (45·7) | 38 (73·1) | 33 (75·0) | 13 (68·4) | 96 (53·6) | 46 (73·0) |  |
| Migration background | |  |  |  |  |  |  |  | - |
|  | Western (including Dutch) | 36 (14·9) | 21 (16·5) | 9 (17·3) | 4 (9·1) | 2 (10·5) | 30 (16·8) | 6 (9·5) |  |
|  | Turkish | 48 (19·8) | 31 (24·4) | 7 (13·5) | 6 (13·6) | 3 (15·8) | 39 (21·8) | 9 (14·3) |  |
|  | Moroccan | 67 (27·7) | 31 (24·4) | 17 (32·7) | 11 (25·0) | 8 (42·1) | 48 (26·8) | 19 (30·2) |  |
|  | Surinamese | 27 (11·2) | 15 (11·8) | 5 (9·6) | 6 (13·6) | 1 (5·3) | 20 (11·2) | 7 (11·1) |  |
|  | Other | 64(26·4) | 29 (22·8) | 14 (26·9) | 17 (38·6) | 5 (26·3) | 42 (23·5) | 22 (34·9) |  |
| Religion | |  |  |  |  |  |  |  | * |
|  | Christianity | 44 (18·2) | 16 (12·6) | 8 (15·4) | 14 (31·8) | 6 (31·6) | 24 (13·4) | 20 (31·7) |  |
|  | Islam | 142 (58·7) | 79 (62·2) | 30 (57·7) | 23 (52·3) | 10 (52·6) | 109 (60·9) | 33 (52·4) |  |
|  | Not religious/ other | 56 (23·1) | 32 (25·2) | 14 (26·9) | 7 (15·9) | 3 (15·8) | 46 (25·7) | 10 (15·9) |  |

^a^For continuous variables the median (IQR) values of the 10th imputed dataset are presented

^b^ISCED 2= Lower secondary education, ISCED 3= Upper secondary education

^c^For differences between food secure and food insecure participants. * indicates a P-value <0.05, P-values were based on Mann-Whitney U test (continuous variables) or Chi-squared test (categorical variables)

Additional Table 4 Potential mediators used in the mediation analyses in the total population and across food insecurity categories

|  | | Total population  (n=242) | High food security (n=127) | Marginal food security (n=52) | Low food security (n=44) | Very low food security (n=19) | Total food secure (n=179) | Total food insecure (n=63) |  |
| --- | --- | --- | --- | --- | --- | --- | --- | --- | --- |
| **Potential mediators**  n (%) or median (IQR) | |  |  |  |  |  |  |  | Significance^c^ (* = p <0.05) |
| Living situation | |  |  |  |  |  |  |  | * |
|  | Married/ partner | 165 (68·2) | 100 (78·7) | 30 (57·7) | 25 (56·8) | 10 (52·6) | 130 (72·6) | 35 (55·6) |  |
|  | Single | 77 (31·8) | 27 (21·3) | 22 (42·3) | 19 (43·2) | 9 (47·4) | 49 (27·4) | 28 (44·4) |  |
| Physical activity (min/ d)^a^ | | 8·6 (2·0, 17·1) | 10·7 (0·7, 17·1) | 9·6 (5·0, 23·0) | 5·7 (1·1, 22·5) | 4·3 (0·0, 12·9) | 10·7 (2·1, 17·1) | 5·0 (0·7, 14·3) | - |
| Household composition (adult/child ratio)^1^ | | 1·0 (0·5, 1·0) | 1·0 (0·7, 1·0) | 0·7 (0·5, 1·0) | 0·7 (0·5, 1·0) | 1·0 (0·7, 2·0) | 1·0 (0·5, 1·0) | 0·7 (0·5, 1·0) | - |
| Smoking status | |  |  |  |  |  |  |  | * |
|  | Smoker | 41 (16·9) | 15 (11·8) | 10 (19·2) | 11 (25·0) | 6 (31·6) | 24 (13·4) | 17 (27·0) |  |
|  | Non-smoker | 201 (83·1) | 112 (88·2) | 42 (80·8) | 33 (75·0) | 13 (68·4) | 155 (86·6) | 46 (73·0) |  |
| General health status^b^ | |  |  |  |  |  |  |  | * |
|  | Good to excellent | 149 (74·9) | 86 (81·1) | 35 (79·5) | 23 (65·7) | 5 (35·7) | 121 (80·7) | 28 (57·1) |  |
|  | Fair to poor | 50 (25·1) | 20 (18·9) | 9 (20·5) | 12 (34·3) | 9 (64·3) | 29 (19·3) | 21 (42·9) |  |
| TOT-Diet score^a^ | | 34·2 (30·8, 41·3) | 34·4 (31·6, 41·3) | 35·9 (31·1, 41·8) | 34·2 (28·5, 39·8) | 31·4 (26·4, 35·3) | 34·7 (31·4, 41·3) | 32·8 (27·2, 38·8) | * |
| FIN-Diet score^a^ | | 17·5 (13·3, 20·9) | 18·2 (14·8, 21·3) | 18·2 (13·5, 20·9) | 14·5 (9·3, 21·1) | 12·3 (6·3, 18·0) | 18·2 (14·6, 21·3) | 13·6 (9·1, 20·0) | * |

^a^For continuous variables the median (IQR) values of the 10th imputed dataset are presented

^b^The question on general health status was added at a later stage during the study and data are therefore missing for 43 (17·8%) participants

^c^For differences between food secure and food insecure participants. * indicates a P-value <0.05, P-values were based on Mann-Whitney U test (continuous variables) or Chi-squared test (categorical variables)

Additional Table 5 Diet quality (component) scores, for the total study population and split by food insecurity status

|  |  | **Score**  median (IQR) | | |  |
| --- | --- | --- | --- | --- | --- |
|  | **Range score** | **Total** | **Food secure** | **Food insecure** | **p-value^a^** |
| **Component** |  |  |  |  |  |
| Vegetables | 0-10 | 4·8 (2·5; 7·5) | 4·8 (2·9; 7·5) | 3·2 (1·6; 7·5) | 0·048* |
| Fruit | 0-10 | 5·9 (2·5; 7·5) | 5·9 (3·8; 7·5) | 3·8 (1·3; 7·5) | 0·001* |
| Fish | 0-10 | 7·5 (5·0; 7·5) | 7·5 (5·0; 7·5) | 7·5 (5·0; 7·5) | 0·007* |
| Bread | 0-10 | 7·5 (6·3; 9·2) | 7·5 (6·3; 8·8) | 7·5 (6·9; 10·0) | 0·342 |
| Oils and fats | 0-10 | 5·0 (5·0; 10·0) | 5·0 (5·0; 10·0) | 7·5 (5·0; 10·0) | 0·857 |
| Sweet and savoury snacks | 0-10 | 5·0 (3·8; 6·3) | 4·6 (3·8; 6·3) | 5·4 (3·8; 7·3) | 0·232 |
| TOT-Diet score | 0-60 | 34·2 (30·8; 41·3) | 34·7 (31·4; 41·3) | 32·8 (27·2; 38·8) | 0·012* |
| FIN-Diet score | 0-30 | 17·5 (13·3; 20·9) | 18·2 (14·6; 21·3) | 13·6 (9·1; 20·0) | <0·001* |

*Statistically significant

^a^P-values based on Mann-Whitney U tests

Additional Table 6 Diet quality (component) scores, in total and split by food insecurity status and obesity status

|  |  | **Score**  median (IQR) | |  |  |
| --- | --- | --- | --- | --- | --- |
|  | **Range score** | **Non-obese** | **Obese** | **p-value^a^** |  |
| **Total population** |  |  |  |  |  |
| Vegetables | 0-10 | 4·8 (2·3; 7·2) | 5·0 (3·0; 8·3) | 0·173 |  |
| Fruit | 0-10 | 5·9 (2·5; 7·5) | 5·9 (2·5; 7·7) | 0·392 |  |
| Fish | 0-10 | 7·5 (5·0; 7·5) | 7·5 (5·0; 7·5) | 0·705 |  |
| Bread | 0-10 | 7·5 (6·3; 8·8) | 6·9 (6·3; 10·0) | 0·878 |  |
| Oils and fats | 0-10 | 5·0 (5·0; 10·0) | 5·0 (5·0; 10·0) | 0·593 |  |
| Sweet and savoury snacks | 0-10 | 5·0 (3·8; 6·8) | 5·0 (3·8; 6·3) | 0·942 |  |
| TOT-Diet score | 0-60 | 33·8 (31·1; 40·3) | 35·3 (29·9; 42·5) | 0·407 |  |
| FIN-Diet score | 0-30 | 17·3 (13·1; 20·3) | 18·2 (13·4; 22·2) | 0·234 |  |
| **Food secure** |  |  |  |  | |
| Vegetables | 0-10 | 4·8 (2·5; 7·1) | 5·5 (3·8; 9·6) | 0·027* | |
| Fruit | 0-10 | 5·9 (3·8; 7·5) | 5·9 (4·4; 7·9) | 0·423 | |
| Fish | 0-10 | 7·5 (5·0; 7·5) | 7·5 (5·0; 7·5) | 0·666 | |
| Bread | 0-10 | 7·5 (6·3; 8·8) | 6·9 (4·7; 10·0) | 0·945 | |
| Oils and fats | 0-10 | 5·0 (5·0; 10·0) | 5·0 (5·0; 10·0) | 0·512 | |
| Sweet and savoury snacks | 0-10 | 4·5 (3·8; 6·4) | 4·8 (3·8; 6·3) | 0·615 | |
| TOT-Diet score | 0-60 | 33·9 (31·4; 40·5) | 37·3 (31·3; 44·0) | 0·171 | |
| FIN-Diet score | 0-30 | 17·5 (14·4; 20·2) | 19·5 (16·1; 22·8) | 0·035* | |
| **Food insecure** |  |  |  |  | |
| Vegetables | 0-10 | 3·2 (1·6; 7·5) | 3·7 (1·6; 7·5) | 0·917 | |
| Fruit | 0-10 | 2·5 (1·1;7·5) | 5·4 (1·5; 7·5) | 0·196 | |
| Fish | 0-10 | 7·5 (5·0; 7·5) | 7·5 (5·0; 7·5) | 0·709 | |
| Bread | 0-10 | 7·5 (6·9; 10·0) | 7·2 (6·4; 10·0) | 0·616 | |
| Oils and fats | 0-10 | 7·5 (5·0; 10·0) | 5·0 (5·0; 9·4) | 0·926 | |
| Sweet and savoury snacks | 0-10 | 5·4 (3·8; 7·3) | 5·5 (3·8; 7·3) | 0·792 | |
| TOT-Diet score | 0-60 | 31·7 (26·5; 37·5) | 34·2 (27·3; 39·8) | 0·761 | |
| FIN-Diet score | 0-30 | 12·5 (9·0; 20·7) | 15·5 (9·4; 20·0) | 0·580 | |

*Statistically significant

^a^P-values based on Mann-Whitney U tests

Additional Table 7 Mediation statistics of the food insecurity status score-obesity association and all potential mediators (unadjusted)

|  | **Indirect effect** | | **Direct effect** | | **Total effect** | | **Proportion of total effect mediated** |
| --- | --- | --- | --- | --- | --- | --- | --- |
|  | Estimate | 95% CI^a^ | Estimate | 95% CI^a^ | Estimate | 95% CI^a^ | % |
| **Mediators** |  |  |  |  |  |  |  |
| Living situation | 0·0367 | 0·00734; 0·0961* | 0·204 | 0·0531; 0·344* | 0·240 | 0·0843; 0·374* | 15·3 |
| Physical activity | 0·00226 | -0·00798; 0·0281 | 0·231 | 0·0909; 0·368* | 0·233 | 0·0907; 0·369* | 0·97 |
| Adult/ child ratio | 0·0102 | -0·00511; 0·0477 | 0·225 | 0·0746; 0·361* | 0·235 | 0·0824; 0·371* | 4·4 |
| Smoking status | -0·0283 | -0·0870; 0·00310 | 0·257 | 0·123; 0·403* | 0·229 | 0·0971; 0·366* | -12·4 |
| General health status | 0·0440 | 0·000891; 0·110* | 0·186 | 0·00406; 0·347* | 0·230 | 0·0633; 0·391* | 19·1 |
| FIN-Diet score | -0·0413 | -0·105;-0·00120* | 0·275 | 0·133; 0·418* | 0·234 | 0·0887; 0·370* | -17·7 |
| TOT-Diet score | -0·0212 | -0·0700; 0·00666 | 0·255 | 0·107; 0·389* | 0·234 | 0·0903; 0·361* | -9·1 |

CI: Confidence Interval

*Statistically significant

^a^Bias-corrected confidence interval

Additional Table 8 Mediation statistics of the food insecurity status score-obesity association and all potential mediators (adjusted^a^)

|  | **Indirect effect** | | **Direct effect** | | **Total effect** | | **Proportion of total effect mediated** |
| --- | --- | --- | --- | --- | --- | --- | --- |
|  | Estimate | 95% CI^b^ | Estimate | 95% CI^b^ | Estimate | 95% CI^b^ | % |
| **Mediators** |  |  |  |  |  |  |  |
| Living situation | 0·0356 | 0·00130; 0·105* | 0·196 | 0·0249; 0·342* | 0·232 | 0·0551; 0·377* | 15·4 |
| Physical activity | 0·00265 | -0·00792; 0·0306 | 0·220 | 0·0441; 0·376* | 0·223 | 0·0496; 0·376* | 1·2 |
| Adult/ child ratio | 0·00548 | -0·00640; 0·0423 | 0·218 | 0·0245; 0·377* | 0·224 | 0·0288; 0·375* | 2·5 |
| Smoking status | -0·0339 | -0·108; -0·000338* | 0·248 | 0·0662; 0·405* | 0·214 | 0·0372; 0·377* | -15·8 |
| General health status | 0·0353 | -0·00502; 0·104 | 0·188 | -0·0271; 0·362 | 0·223 | 0·0104; 0·395* | 15·8 |
| FIN-Diet score | -0·0418 | -0·102; -0·00185* | 0·267 | 0·0756; 0·409* | 0·225 | 0·0398; 0·370* | -18·6 |
| TOT-Diet score | -0·0206 | -0·0714; 0·00289 | 0·245 | 0·0519; 0·397* | 0·225 | 0·0257; 0·370* | -9·2 |

CI: Confidence Interval

*Statistically significant

^a^Adjusted for age, sex, household income, educational level, and migration background

^b^Bias-corrected confidence interval
